# Eligibility for HIV Preexposure Prophylaxis, Intention to Use Preexposure Prophylaxis, and Informal Use of Preexposure Prophylaxis Among Men Who Have Sex With Men in Amsterdam, the Netherlands

**DOI:** 10.1097/OLQ.0000000000001291

**Published:** 2020-09-15

**Authors:** Sebastiaan H. Hulstein, Amy Matser, Maarten F. Schim van der Loeff, Elske Hoornenborg, Maria Prins, Henry J.C. de Vries

**Affiliations:** From the ∗ Department of Infectious Diseases, Public Health Service of Amsterdam; † Division of Infectious Diseases, Department of Internal Medicine; ‡ Department of Dermatology, University of Amsterdam, and Amsterdam Institute for Infection and Immunity (AI&II), location Academic Medical Centre, Amsterdam, the Netherlands

## Abstract

**Background:**

Before 2019, availability of affordable HIV preexposure prophylaxis (PrEP) was limited in the Netherlands. Consequently, some men who have sex with men and transgender persons obtained PrEP informally, that is, without a prescription.

**Methods:**

Between September 2017 and August 2018, we enrolled informal PrEP users in a cohort study (Informal PrEP [InPrEP]) at the Amsterdam Sexually Transmitted Infections (STI) Clinic. We compared PrEP use, PrEP eligibility and intention, chemsex, and STI positivity between 3 groups: (1) informal PrEP users enrolled in InPrEP, (2) nonenrolled informal PrEP users, and (3) non-PrEP users. Also, we assessed intention and associated characteristics among non-PrEP users who were eligible for PrEP.

**Results:**

During the study period, we enrolled 118 informal PrEP users in InPrEP who were compared with 224 nonenrolled informal PrEP users and 4975 non-PrEP users. Preexposure prophylaxis eligibility rates were 83.1%, 93.4%, and 63.0%; chemsex were reported by 39.1%, 48.1%, and 11.7%; and STI positivity was reported by 28.0%, 42.5%, and 20.3%, respectively. Among PrEP-eligible non-PrEP users, PrEP intention was 16.6% and associated with increasing age, non-Dutch origin, higher educational level, receptive condomless anal sex, increasing number of sex partners, chemsex, and sex with men only.

**Conclusions:**

Most informal PrEP users are eligible to use PrEP and have a clear sexual health care need. Especially in settings with limited PrEP access, providing the necessary care for informal PrEP users is important. The intention to use PrEP is low among PrEP-eligible STI clinic visitors. Further research should investigate modes to increase PrEP intention among PrEP-eligible persons.

## Abstract 2

A cross-sectional study showed that informal preexposure prophylaxis users visiting the Amsterdam Sexually Transmitted Infections Clinic are in need of regular (sexual) health care checkups.

Supplemental digital content is available in the text.

In the Netherlands, 65.8% of the newly diagnosed HIV infections in 2018 occurred among men who have sex with men (MSM), indicating a focused epidemic.^1^ The Netherlands have achieved the Joint United Nations Programme on HIV/AIDS 90-90-90 goals, which targets to identify at least 90% of persons living with HIV, treat at least 90% of those diagnosed, and achieve viral suppression in >90% of treated cases.^1,2^ However, increased efforts are needed to halt HIV spread. Increased uptake of preexposure prophylaxis (PrEP) may steer the HIV epidemic closer toward zero.

However, despite evidence of efficacy, cost-effectiveness, and safety of PrEP, national implementation of PrEP has been piecemeal in many countries for various reasons,^3^ including costs and the question of who should pay for PrEP.^4–6^ In settings of limited or rationed access to formal PrEP, the use of informally accessed generic PrEP (i.e., without a prescription) has emerged^7–9^ and may continue in countries (e.g., Austria and Macedonia) with inadequate PrEP availability up to this date.^10^ Moreover, in several countries where same-gender relationships are a criminal offense or heavily stigmatized,^11^ informal PrEP use may be the only available option to access PrEP. Recent evidence suggests that informal PrEP use is associated with suboptimal STI and HIV testing before and during PrEP use, which may lead to resistant HIV infections and ongoing transmission.^12^

Not much is known about informal PrEP in the Netherlands and about eligibility to use PrEP among informal PrEP users in general. In addition, data on other identifiers of sexual health care needs, such as chemsex (the use of γ-hydroxybutyric acid, mephedrone, or crystalized methamphetamine in the direct context of sex),^13^ are also limited among informal PrEP users but are required to direct public health interventions and to organize health care.^3,7,9^ In the Netherlands, a national PrEP program was not yet in place until August 2019. Therefore, we started a health care program at the Amsterdam Sexually Transmitted Infections (STI) Clinic for informal PrEP users. In addition, informal PrEP users were invited to enroll in the Informal PrEP (InPrEP) study, a prospective cohort that was nested in the health care program and aimed to study their characteristics and sexual health care needs.

The primary aims of the current study were the following: (1) to describe sociodemographics, sexual behavior, chemsex, and STI prevalence of informal PrEP users enrolling in a prospective cohort study; (2) to compare these with informal PrEP users not enrolling in the prospective cohort study and with non-PrEP users; and (3) to assess PrEP eligibility (according to Dutch guidelines) of informal PrEP users and non-PrEP users. The secondary aims were (1) to assess intention to use PrEP among PrEP-eligible non-PrEP users and identify characteristics associated with a high intention to use PrEP, and (2) to assess associations with informal PrEP use.

## METHODS

### Study Population

HIV-negative MSM and transgender persons (TGPs) who attended the Amsterdam STI Clinic between September 27, 2017, and August 2, 2018, and were at least 18 years of age were invited to participate in InPrEP, if they reported informal PrEP use in the past 3 months, without further exclusion criteria for participation.

Informal PrEP use was defined as the acquisition and use of formulations of tenofovir/emtricitabine in the 3 months before the clinic visit without a prescription of a health care worker within the Dutch health care system, or through PrEP clinical trials or demonstration studies. InPrEP is a prospective cohort study that aims to provide PrEP care and study characteristics, PrEP use and adherence, sexual behavior, chemsex, and STI among informal PrEP users. Informal PrEP participants were enrolled on the same day as their routine clinic visit in which they reported PrEP, or within 4 weeks after the first visit, if logistical constraints of the STI clinic prevented enrollment on the same day.

**Figure 1:**
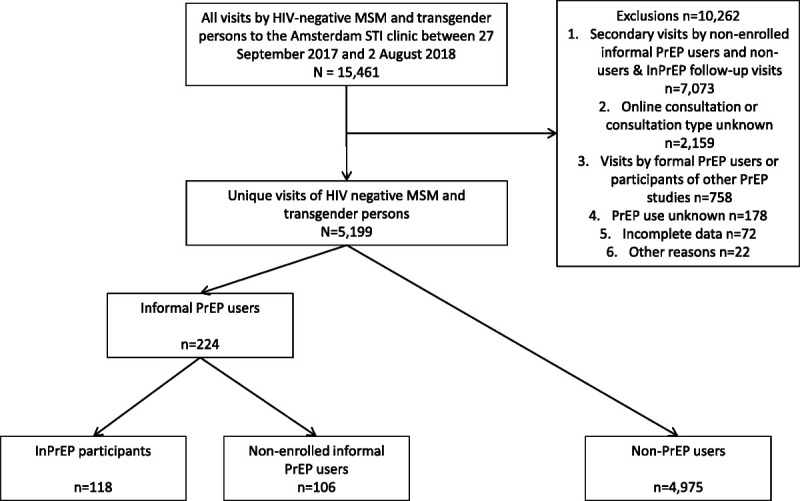
Flow chart and reasons for inclusion and exclusion of InPrEP participants, informal PrEP users outside the study and non- users of PrEP who visited the Amsterdam STI clinic between 27 September 2017 and 2 August 2018.

In this cross-sectional analysis, we compared the (1) baseline characteristics of informal PrEP users enrolled in InPrEP with (2) the characteristics of informal PrEP users who declined participation in InPrEP (i.e., nonenrolled informal PrEP users) and (3) the characteristics of individuals who had not used PrEP in the past 3 months (i.e., non-PrEP users; Fig. 1). In addition, we ascertained the intention to use PrEP and its determinants among non-PrEP users who are eligible to use PrEP according to the Dutch PrEP guidelines (see details hereinafter).^14^ Finally, we assessed the associations with informal PrEP use among all aforementioned groups.

For nonenrolled informal PrEP users and non-PrEP users with multiple visits in the study period, the first visit with complete data was used. We excluded formal PrEP users and those who obtained PrEP through other studies from the analysis.

The InPrEP study was reviewed by the ethics board of the Amsterdam UMC, location Academic Medical Center, and waived the necessity of a full review (letter no.: W17_015 #17.052). Nevertheless, written informed consent was obtained from participants.

### HIV/STI Testing

All HIV-negative MSM and TGP Amsterdam STI Clinic clients were offered routine STI testing at their visit for *Chlamydia trachomatis*, *Neisseria gonorrhoeae* (Aptima Combo 2, Hologic Gen-Probe Inc., San Diego, CA) on urine and rectal and pharyngeal samples, and for syphilis, HIV, and hepatitis B virus on venous blood samples. In addition, InPrEP participants were tested for hepatitis C virus infection (enzyme-linked immunosorbent assay: anti–hepatitis C virus) and serum creatinine at 3-monthly study visits in accordance with the Dutch PrEP guidelines.^14^ Participants of the InPrEP study who returned within 4 weeks for enrollment after their initial visit were retested for chlamydia, gonorrhea, syphilis, and HIV if STI-related symptoms occurred.

### Data Collection

Data on sociodemographics, PrEP use and intention, access route of PrEP, sexual behavior, chemsex, self-reported symptoms attributed to STI, and sex partner notification for STI were collected during the STI Clinic visit as part of routine care and recorded in the electronic patient file. For InPrEP participants with a return visit within 4 weeks because of logistical constraints, we used the data from the return visit. Data on STI positivity were taken from the preceding visit if no STI tests were performed during the return visit. In addition, InPrEP participants completed a study questionnaire on PrEP use and adherence, detailed sexual behavior, chemsex, and psychosocial outcomes.

### Variables

#### Sociodemographics

Age, self-reported gender (assessed using the 2-step method for determining the sex assigned at birth and gender identity^15^), ethnicity (based on the country of birth of the person and their parents^16^ and classified as the Netherlands; North Africa, the Near- and Middle East; Middle and South America; Europe; and other non-European), and educational level were assessed. Educational level was categorized as low (no education, primary school, or lower secondary education), medium (intermediate or higher secondary education, higher secondary vocational education), high (college or university), and other.

#### PrEP Variables

Preexposure prophylaxis eligibility was assessed according to the Dutch PrEP guidelines.^14^ Any HIV-negative MSM or TGP who met at least 1 of the following criteria in the past 6 months was eligible for PrEP use: (1) engaged in condomless anal sex with a male or transgender partner with an unknown HIV status or an HIV positive status with a detectable viral load, or (2) was diagnosed with a rectal bacterial STI, or (3) had received postexposure prophylaxis for a sexual exposure incident.

The PrEP dosing regimen used in the past 3 months was categorized as strictly daily, strictly event-driven, daily and event-driven PrEP use, and other. Event-driven use was defined as taking 2 tablets 2 to 24 hours before sex, followed by 1 tablet 24 hours after the first dose, and 1 tablet 48 hours after the first dose, as described earlier.^17^

Preexposure prophylaxis intention was measured among individuals who were not on PrEP using the following question: “Do you have the intention to start PrEP in the coming 30 days?” The answer “yes” was classified as a “high” intention, and “no” was classified as “low.”

Preexposure prophylaxis access route was assessed by asking the source of any acquired PrEP and was categorized as follows: via acquaintance, online, buyers' club, on travel, and other. We defined a buyers' club as a group of individuals who have organized to buy PrEP collectively, subsequently lowering the price for the individual members of the group.

#### Sexual Behavior and Chemsex

Sexual behavior was assessed as the self-reported number of sex partners categorized in quartiles and anal sex practices in the past 6 months (with or without a condom and insertive or receptive position). We assessed the self-reported use of γ-hydroxybutyric acid, crystalized methamphetamine, and mephedrone separately and defined chemsex as the self-reported use of at least 1 of the aforementioned substances in the context of sex in the past 6 months.

### Statistical Analyses

For all analyses, we used the data from the enrollment visit for InPrEP participants and the first visit with complete data for nonenrolled informal PrEP users and non-PrEP users. We described the characteristics, including PrEP eligibility, of the InPrEP study participants and compared these WITH those of nonenrolled informal PrEP users and non-PrEP users. We used χ^2^ tests for categorical data and Kruskal-Wallis tests for continuous data to test for statistically significant differences between groups.

Second, we described the intention to use PrEP among non-PrEP users who are eligible to use PrEP according to the Dutch PrEP guidelines.^14^ We assessed factors associated with a high intention to use PrEP using univariable and multivariable logistic regression analyses. In addition, we assessed factors associated with informal PrEP use among the 3 groups using univariable and multivariable logistic regression analyses. Factors of interest were sociodemographics, sexual behavior, chemsex, and STI diagnoses. In these analyses, age and gender were forced into the model as potential confounders. *P* < 0.05 was considered statistically significant. All analyses were performed in STATA Intercooled 15.1 (STATA Corporation, College Station, TX).

## RESULTS

A total of 5199 singular visits by 5119 HIV-negative MSM and 80 TGP between September 27, 2017, and August 2, 2018, were included in the analysis (Fig. 1). Informal PrEP use was reported by 224 of (4.3%) 5199 clients, of whom 118 enrolled in InPrEP. Overall, 3333 (64.1%) of 5199 clients were eligible for PrEP use according to the Dutch guidelines.

### Comparison of PrEP Eligibility, and Baseline Characteristics Of InPrEP Participants (n = 118), Nonenrolled Informal PrEP Users (n = 106), and Non-PrEP Users (n = 4975)

Among InPrEP participants, nonenrolled informal PrEP users, and non-PrEP users, 98 (83.1%) of 118, 99 (93.4%) of 106, and 3136 (63.0%) of 4975 were eligible for PrEP according to the Dutch PrEP guidelines (*P* < 0.001; Table 1).

**TABLE 1:** Baseline Characteristics of InPrEP Participants Compared With Nonenrolled Informal PrEP Users and Non-PrEP Users Who Visited the Amsterdam STI Clinic Between September 27, 2017, and August 2, 2018

	Missing	InPrEP Participants (n = 118)		Nonenrolled Informal PrEP Users (n = 106)		Non-PrEP Users (n = 4975)		
	n	n/M	%/(IQR)	n/M	%/(IQR)	n/M	%/(IQR)	*P*
General								
Gender	0							0.723
Male		118	99.2%	105	99.1%	4897	98.4%	
Transgender*		1	0.8%	1	0.9%	78	1.6%	
Median age (IQR), y	0	40	(34–47)	33	(28–39)	31	(25–40)	<0.001
Country or region of origin	4							<0.001
The Netherlands		68	57.6%	46	43.4%	2452	49.3%	
North Africa, the Near- and Middle East^†^		13	11.0%	20	18.9%	771	15.5%	
Middle and South America^‡^		6	5.1%	13	12.3%	619	12.5%	
Europe^§^		20	17.0%	15	14.2%	906	18.2%	
Other non-European^¶^		11	9.3%	12	11.3%	225	4.5%	
Educational level	147							0.379
Low		3	2.6%	7	6.6%	247	5.1%	
Medium		16	14.0%	14	13.2%	870	17.8%	
High		85	74.6%	71	67.0%	3196	65.5%	
Other		10	8.8%	14	13.2%	570	11.7%	
PrEP								
PrEP eligibility	0	98	83.1%	99	93.4%	3136	63.0%	<0.001
CAS with sex partner of unknown/detectable HIV status		97	82.2%	99	93.4%	3115	62.6%	<0.001
Rectal STI in past 6 mo		15	12.7%	14	13.2%	265	5.3%	<0.001
PEP course in past 6 mo		4	3.4%	5	4.7%	67	1.4%	0.004
PrEP access route, users only	22							<0.001
Via acquaintance		14	13.2%	33	33.3%	N/A		
Online		52	49.1%	32	32.3%	N/A		
Buyers’ club		32	30.2%	9	9.1%	N/A		
On travel		5	4.7%	22	22.2%	N/A		
Other^∥^		3	2.8%	3	3.0%	N/A		
Use of PrEP regimen (users only)	10					N/A		0.037
Strictly daily		52	46.9%	43	41.4%	N/A		
Strictly event-driven		49	44.1%	50	48.1%	N/A		
Daily and event-driven		10	9.0%	4	3.9%	N/A		
Other**		0	0.0%	5	6.7%	N/A		
Sexual behavior and chemsex								
Sex with								<0.001
Men and women		0	0	4	3.8%	768	15.5%	
Men only		118	100%	102	96.2%	4207	84.5%	
Anal sex in past 6 mo	70							<0.001
No anal sex		1	0.9%	0	0%	399	8.1%	
Yes, only with a condom		16	14.0%	5	4.8%	1405	28.6%	
Yes, insertive without condom, but no receptive sex or with condom		7	6.1%	12	11.5%	877	17.8%	
Yes, receptive without condom, regardless of insertive sex		90	79.0%	87	83.7%	2238	45.5%	
Median no. sex partners in past 6 mo (IQR)	40	15	(6–30)	15	(6–30)	6	(3–10)	<0.001
STI-related symptoms at the time of consultation	4	4	3.4%	33	68.9%	1087	21.9%	<0.001
Notified by sex partner for bacterial STI	8	7	6.0%	34	32.1%	1202	24.2%	<0.001
Chemsex in past 6 mo	83	45	39.1%	51	48.1%	576	11.7%	<0.001
γ-Hydroxybutyric acid^††^		41	34.8%	48	45.3%	444	8.9%	<0.001
Methylamphetamine^††^		9	7.6%	17	33.3%	65	11.3%	<0.001
Mephedrone^††^		6	5.1%	8	7.6%	27	0.5%	<0.001
STI								
Any STI		33	28.0%	45	42.5%	1013	20.3%	<0.001
Chlamydia, anal	2	7	6.0%	17	16.0%	325	6.5%	0.001
Chlamydia, any site	2	14	12.0%	21	19.8%	460	9.3%	0.001
LGV (anal or inguinal)	2	2	1.7%	3	2.8%	8	0.2%	<0.001
Gonorrhea, anal	2	9	7.7%	21	19.8%	360	7.2%	<0.001
Gonorrhea, any site	2	18	15.4%	25	23.6%	583	11.7%	0.001
Infectious syphilis (stage 1, 2, or early latent)	0	5	4.2%	9	8.5%	101	2.0%	<0.001
Late (latent) syphilis	0	0	0%	0	0%	44	0.9%	0.368

InPrEP participants are HIV-negative MSM and TGP who visited the Amsterdam STI Clinic between September 27, 2017, and August 2, 2018 and self-reported informal PrEP use in the past 3 months and enrolled in InPrEP. Informal PrEP users outside the study are HIV-negative MSM and TGP who visited the Amsterdam STI Clinic between September 27, 2017, and August 2, 2018 and self-reported informal PrEP use in the past 3 months and did not enroll in InPrEP. Nonusers of PrEP are HIV-negative MSM and TGP who did not report PrEP use and visited the STI clinic between September 27, 2017, and August 2, 2018.

*All transgender persons self-identified as transgender women.

^†^Including Turkey and Morocco.

^‡^Including Dutch Antilles and Suriname.

^§^All countries from the European Region, except for the Netherlands.

^¶^Including North America and Sub-Saharan Africa.

^∥^Other category consists of acquired via HIV-positive partner, via PEP course, via other means, and 2 or more sources.

**Other category consists of unknown and regimens without evidence.

^††^Provided the person had engaged in sexualized substance use in the past 6 months.

CAS indicates condomless anal sex; IQR, interquartile range; LGV, lymphogranuloma venereum; M, median; n, number; PEP, postexposure prophylaxis; PrEP, preexposure prophylaxis; STI, sexually transmitted infections.

The median age of InPrEP participants was 40 years (interquartile range [IQR], 34–47 years). They were older than the nonenrolled informal PrEP users (M: 33 years, IQR 28–39), and older than non-PrEP users (median, 31 years; IQR, 25–40 years). Informal PrEP participants were more often Dutch than nonenrolled informal PrEP users and non-PrEP users (57.6% vs. 43.4% and 49.3%, respectively). Overall, educational level was high and did not differ across groups.

Self-reported PrEP regimen differed between InPrEP participants and nonenrolled informal PrEP users, specifically the “daily and event-driven use” (9% vs. 3.9%) and “other” regimen (0% vs. 6.7%).

Receptive condomless anal sex was frequently reported: 79.0% among InPrEP participants, 83.7% among nonenrolled informal PrEP users, and 45.5% among non-PrEP users (*P* < 0.001). Also notable, InPrEP participants and nonenrolled informal PrEP users reported more often sex with men only compared with non-PrEP users (100%, 96.2%, and 84.5%, respectively; *P* < 0.001). Chemsex was reported by 39.1% of InPrEP participants, 48.1% of nonenrolled informal PrEP users, and 11.7% of non-PrEP users.

Being diagnosed with any STI was 28.0% among InPrEP participants, 42.5% among nonenrolled informal PrEP users, and 20.3% among non-PrEP users.

### Associations With a High Intention to Use PrEP Among Non-PrEP Users

Of the 3136 non-PrEP users who were eligible for PrEP according to the Dutch PrEP guidelines, 522 (16.6%) had a high intention to use PrEP. A higher age, being diagnosed with a rectal STI in the past 6 months, having sex with men only, receptive condomless anal sex, higher number of sex partners, chemsex, γ-hydroxybutyric acid use, and any STI were associated with a high intention to use PrEP (Table 2).

**TABLE 2:** Associations With a High Intention to Use PrEP Among Nonusers of PrEP Who Were Eligible to Use PrEP According to the Dutch PrEP Guidelines

	Missing	High Intention		Univariable Logistic Regression			Multivariable Logistic Regression		
	n	n/M	%/(IQR)	OR	95% CI	*P*	aOR	95% CI	*P*
General									
Intention*	0	522/3136	16.6%	—	—	—	—	—	—
Gender	0					0.221		0.455	
Male		515/3109	16.6%	1			1		
Transgender*		7/27	25.9%	1.76	(0.74–4.19)		1.44	(0.57–3.66)	
Median age (IQR)^†,‡^, y	0	33	(27–42)	1.18	(1.09–1.27)	<0.001	1.16	(1.06–1.27)	0.001
Country or region of origin	2					0.052			0.004
The Netherlands		231/1536	15.0%	1			1		
North Africa, the Near- and Middle East^§^		84/500	16.8%	1.14	(0.87–1.50)		1.36	(1.01–1.82)	
Middle and South America^¶^		76/393	19.3%	1.35	(1.02–1.80)		1.64	(1.20–2.25)	
Europe (other)^∥^		110/556	19.8%	1.39	(1.08–1.79)		1.53	(1.17–2.01)	
Other non-European**		21/149	14.1%	0.93	(0.57–1.50)		1.09	(0.65–1.82)	
Educational level	65					0.107			0.034
Low		32/184	17.4%	1			1		
Medium		97/564	17.2%	0.99	(0.64–1.53)		1.10	(0.69–1.75)	
High		334/1967	17.0%	0.97	(0.65–1.45)		1.07	(0.70–1.63)	
Other		43/356	12.1%	0.65	(0.40–1.07)		0.64	(0.38–1.09)	
PrEP eligibility	0	N/A	N/A	N/A	N/A	N/A			
CAS with sex partner of unknown/detectable HIV status^††^		517/3115	16.6%	0.64	(0.23–1.75)	0.400			
Rectal STI in past 6 mo^††^		44/191	23.0%	1.54	(1.09–2.19)	0.019			
PEP course in past 6 mo^††^		17/67	25.4%	1.73	(0.99–3.02)	0.067			
Sexual behavior and sexualized drug use									
Sex with	0					<0.001			<0.001
Men and women		23/427	5.4%	1			1		
Men only		499/2709	18.4%	3.97	(2.58–6.10)		4.06	(2.56–6.43)	
Anal sex in past 3 mo	60					<0.001			0.004
No anal sex or with condom only		5/21	23.8%	2.13	(0.77–5.94)		2.09	(0.71–6.16)	
Yes, insertive without condom, receptive sex only with condom		112/877	12.8%	1			1		
Yes, receptive without condom		405/2238	18.1%	1.51	(1.20–1.89)		1.49	(1.17–1.91)	
No. sex partners in past 6 mo	11					<0.001			<0.001
1–3 sex partners		86/881	9.8%	1			1		
4–6 sex partners		128/890	14.4%	1.55	(1.16–2.08)		1.71	(1.26–3.21)	
7–10 sex partners		95/592	16.0%	1.77	(1.29–2.42)		1.81	(1.30–2.52)	
11+ sex partners		212/762	27.8%	3.56	(2.71–4.68)		3.89	(2.52–4.55)	
STI-related symptoms at the time of consultation^††^		109/721	15.1%	0.86	(0.69–1.09)	0.206			
Notified by sex partner for bacterial STI^††^	2	127/788	16.1%	0.95	(0.76–1.18)	0.658			
Chemsex in past 6 mo^††^	34	131/457	28.7%	2.35	(1.87–2.96)	<0.001	1.85	(1.45–2.37)	<0.001
γ-Hydroxybutyric acid^††,‡‡^		113/359	31.5%	2.04	(1.16–3.57)	0.009			
Methylamphetamine^††,‡‡^		19/58	32.8%	1.25	(0.69–2.25)	0.466			
Mephedrone^††,‡‡^		10/24	41.7%	1.84	(0.80–4.26)	0.162			
STI									
Any STI^††^	0	140/732	19.1%	1.25	(1.01–1.55)	0.042			
Chlamydia, anal^††^	0	45/245	18.4%	1.14	(0.81–1.60)	0.449			
Chlamydia, any site^††^	0	64/342	18.7%	1.18	(0.88–1.57)	0.277			
LGV (anal or inguinal)^††^	0	2/6	33.3%	2.51	(0.46–13.76)	0.318			
Gonorrhea, anal^††^	0	56/272	20.6%	1.34	(0.98–1.82)	0.073			
Gonorrhea, any site^††^	0	82/413	19.6%	1.29	(0.99–1.67)	0.063			
Infectious syphilis (stage 1, 2, or early latent)^††^	0	12/78	15.4%	0.91	(0.49–1.69)	0.760			
Late (latent) syphilis^††^	0	9/30	30.0%	2.17	(0.99–4.76)	0.069			

Nonusers of PrEP are HIV-negative MSM and TGP who did not report PrEP use and visited the STI clinic between September 27, 2017, and August 2, 2018.

*All transgender persons self-identified as transgender women.

^†^The median age of non-PrEP users with a low intention to use PrEP is 30 years (IQR, 25–39 years).

^‡^The OR and aOR represent the association of the median age in increments of 10 years with a high intention to use PrEP.

^§^Including Turkey and Morocco.

^¶^Including Dutch Antilles and Suriname.

^∥^All countries from the European Region, except for the Netherlands.

* Including North America and Sub-Saharan Africa.

^††^For logistic regression analyses, the reference variable was the absence of the condition.

^‡‡^Provided the participant had engaged in sexualized drug use in the past 6 months.

aOR indicates adjusted odds ratio; CAS, condomless anal sex; CI, confidence interval; IQR, LGV, lymphogranuloma venereum; interquartile range; M, median; n, number; OR, odds ratio; *P*, probability; PEP, postexposure prophylaxis; PrEP, preexposure prophylaxis; STI, sexually transmitted infection.

Multivariable logistic regression analyses (Table 2) showed that, adjusting for age and gender, non-Dutch country or region of birth, higher educational level, sex with men only, receptive condomless anal sex, higher number of sex partners, and chemsex were all associated with a high intention to use PrEP.

### Associations With Informal PrEP Use

An increasing age, being of Dutch origin, being diagnosed with a rectal STI in the past 6 months, receptive condomless anal sex, higher number of sex partners, chemsex, and being diagnosed with infectious syphilis were associated with informal PrEP use in multivariable logistic regression analyses (Supplement 1, http://links.lww.com/OLQ/A555).

## DISCUSSION

Among informal PrEP users enrolled in the InPrEP study, nearly 85% were eligible for PrEP according to the Dutch guidelines, and PrEP eligibility was slightly higher among nonenrolled informal PrEP users. Chemsex and diagnosis of any STI were high among InPrEP participants and nonenrolled informal PrEP users. These results are indicative of a need for the availability of sexual health care for informal PrEP users. Among non-PrEP users who were eligible to use PrEP, approximately 1 of 6 had a high intention to use PrEP. A high intention to use PrEP was associated with a higher educational level, higher number of sex partners, more often reported condomless anal sex, and chemsex, but also with having sex with men only, and being born abroad.

Aloysius et al.^7^ describe a group of informal PrEP users from London who accessed a tenofovir/emtricitabine drug level monitoring service in 2016 to 2017, who were, in comparison with InPrEP participants, of similar age (median, 37 years; IQR, 32–45 years), had fewer STI diagnoses (16% vs. 26.8% in InPrEP), used more often daily PrEP (75% vs. 46.9%), and reported more often use of metamphetamine (14% vs. 7.6% in InPrEP) and mephedrone use (9% vs. 5.1% in InPrEP). In the Netherlands, the use of ecstasy is more common than mephedrone,^18^ which may account for the observed differences in drug use. Moreover, event-driven use of PrEP has been offered in the Dutch Amsterdam PrEP demonstration study^19^ from 2015 onward, which may have resulted in an increased awareness and use of event-driven PrEP.

In the current study, approximately 1 of 6 non-PrEP users who were eligible for PrEP had a high intention to use PrEP. The willingness to use PrEP varies worldwide, with estimates up to 96.2%.^20^ Earlier studies of PrEP intention among MSM participating in the Amsterdam Cohort Studies reported a high intention in 13% in 2012^21^ and in 30% in 2015.^22^ The definition of high PrEP intention in the current study reflects an expectation to initiate PrEP within 30 days, which may underestimate the long-term intention to use PrEP, measured in aforementioned studies. In addition, some persons who had a high intention to use PrEP in earlier studies likely have now started PrEP, which could decrease current estimates of intention. Bil et al.^21^ reported potential barriers of PrEP intention: shame of using PrEP and fear of adverse effects, but these barriers were studied in a period of even more limited PrEP use, awareness, and availability. Further efforts should be made to study and address the barriers of PrEP-eligible MSM and TGP in the current setting of increased PrEP awareness and availability in the Netherlands.

Preexposure prophylaxis use intention was associated with reporting sex with men only. It is unknown why men and TGP who also have sex with women have lower intention to use PrEP. Factors that may explain this disparity include a relatively lower risk perception, awareness, knowledge or the feeling that PrEP is not indicated for them because current PrEP campaigns in the Netherlands are directed to gay-identifying MSM, and not to persons with other gender identities. In future campaigns, special attention for these subgroups is warranted.

The setting of the current study, the Amsterdam STI Clinic, may have influenced the generalizability of our findings. It is very likely that sexual behavior, chemsex and subsequently STI diagnoses, and PrEP eligibility may be higher in this population than in other settings in the Netherlands. However, differences between informal PrEP users and non-PrEP users will be similar in other contexts as well as associations with intentions to use PrEP, as these are independent from the actual numbers.

The current study has certain limitations. First, the classification of PrEP eligibility based on the clients' electronic file is not perfect because the HIV status of the partner was not known, which may have underestimated current estimates of PrEP eligibility. Second, STI positivity rates may be inflated among InPrEP participants because these were taken from the return visit within 4 weeks if symptoms were reported during this visit.

A strength of our study is that we describe informal PrEP users, which is rarely done. The high level of PrEP eligibility, the high proportion of men with STI diagnoses, and chemsex show a clear need for sexual health care for informal PrEP users. This is further underlined by recent findings from Koppe et al.,^12^ showing that informal PrEP use is associated with suboptimal STI, HIV, and renal function testing before and during periods of informal PrEP use. The attention of public health organizations for the specific health care needs for this subgroup of PrEP users is urgently warranted.

In conclusion, this study shows that many people seeking informal PrEP are PrEP eligible according to current PrEP guidelines. Our data show a clear need for sexual health care of informal users, illustrated by their eligibility for PrEP use, chemsex, and high numbers of diagnosed STI. Countries where access to PrEP is limited should seek ways to offer sexual health care for persons who use PrEP informally and strive to make PrEP and related sexual health care available. In addition, a substantial group of PrEP-eligible persons, especially bisexual MSM and TGP, do not opt for PrEP or have an intention to do so, for reasons yet unknown. More research should be directed to uncover the reasons why, so enhanced efforts can be made to motivate these subgroups to use PrEP or other methods of reliable HIV prevention.

## References

[1] van Sighem AWF Boyd A Smit C, HIV Monitoring Report 2019. Amsterdam: Stichting HIV Monitoring (SHM), 2019.

[2] HIV/AIDS JUNPo 90-90-90: An Ambitious Treatment Target to Help End the AIDS Epidemic. Geneva, Switzerland: UNAIDS, 2014.

[3] Hankins C Macklin R Warren M Translating PrEP effectiveness into public health impact: Key considerations for decision-makers on cost-effectiveness, price, regulatory issues, distributive justice and advocacy for access. J Int AIDS Soc 2015; 18(4 Suppl 3):19973. 26198343 10.7448/IAS.18.4.19973 PMC4509900

[4] Nichols BE Boucher CAB van der Valk M, Cost-effectiveness analysis of pre-exposure prophylaxis for HIV-1 prevention in the Netherlands: A mathematical modelling study. Lancet Infect Dis 2016; 16:1423–1429. 27665989 10.1016/S1473-3099(16)30311-5

[5] Cambiano V Miners A Dunn D, Cost-effectiveness of pre-exposure prophylaxis for HIV prevention in men who have sex with men in the UK: A modelling study and health economic evaluation. Lancet Infect Dis 2018; 18:85–94. 29054789 10.1016/S1473-3099(17)30540-6 PMC5988036

[6] van de Vijver DAMC Richter AK Boucher CAB, Cost-effectiveness and budget effect of pre-exposure prophylaxis for HIV-1 prevention in Germany from 2018 to 2058. Euro Surveill 2019; 24:1800398. 10.2807/1560-7917.ES.2019.24.7.1800398 PMC6381659 30782266

[7] Aloysius I Savage A Zdravkov J, InterPrEP. Internet-based pre-exposure prophylaxis with generic tenofovir DF/emtricitabine in London: An analysis of outcomes in 641 patients. J Virus Erad 2017; 3:218–222. 29057086 10.1016/S2055-6640(20)30317-4 PMC5632549

[8] Group FES Flash! PrEP in Europe. Paris, France: AIDES & Coalition Plus, 2016.

[9] Brisson J Ethical public health issues for the use of informal PrEP. Glob Public Health 2018; 13:1382–1387. 28868979 10.1080/17441692.2017.1373139

[10] Aids Vaccine Advocacy Coalition (AVAC) Full list of countries with current PrEP programs. Available at: www.prepwatch.org. Accessed 30 July 2020.

[11] Human rights watch. LGBT rights. Available at: http://internap.hrw.org/features/features/lgbt_laws/. Accessed 21 February 2020.

[12] Koppe U Marcus U Albrecht S, Factors associated with the informal use of HIV pre-exposure prophylaxis in Germany: A cross-sectional study. J Int AIDS Soc 2019; 22:e25395. 31583823 10.1002/jia2.25395 PMC6776824

[13] Druckler S van Rooijen MS de Vries HJC Chemsex among men who have sex with men: A sexualized drug use survey among clients of the sexually transmitted infection outpatient clinic and users of a gay dating app in Amsterdam, the Netherlands. Sex Transm Dis 2018; 45:325–331. 29465683 10.1097/OLQ.0000000000000753 PMC5908259

[14] Nederlandse Vereniging van Hiv Behandelaren HIV Pre-expositie profylaxe (PrEP) richtlijn Nederland 2019 Available at: https://nvhb.nl/wp-content/uploads/2019/04/PrEP-richtlijn-Nederland-versie-2-dd-15-april-2019.pdf. Accessed October 12, 2020.

[15] Deutsch MB Green J Keatley J, Electronic medical records and the transgender patient: Recommendations from the World Professional Association for Transgender Health EMR Working Group. J Am Med Inform Assoc 2013; 20:700–703. 23631835 10.1136/amiajnl-2012-001472 PMC3721165

[16] Stronks K Kulu-Glasgow I Agyemang C The utility of’country of birth’ for the classification of ethnic groups in health research: The Dutch experience. Ethn Health 2009; 14:255–269. 19052941 10.1080/13557850802509206

[17] Molina JM Capitant C Spire B, On-demand preexposure prophylaxis in men at high risk for HIV-1 infection. N Engl J Med 2015; 373:2237–2246. 26624850 10.1056/NEJMoa1506273

[18] Group DMS Nationale Drug Monitor 2018 Available at: https://www.trimbos.nl/kennis/feiten-cijfers-drugs-alcohol-roken/ndm-monitoring-drugsgebruik-in-nederland. Accessed October 12, 2020.

[19] Hoornenborg E Achterbergh RC van der Loeff MFS, Amsterdam PrEP Project team in the HIV Transmission Elimination AMsterdam Initiative Men who have sex with men more often chose daily than event-driven use of pre-exposure prophylaxis: Baseline analysis of a demonstration study in Amsterdam. J Int AIDS Soc 2018; 21:e25105. 29603900 10.1002/jia2.25105 PMC5878413

[20] Niderost S Gredig D Hassler B, The intention to use HIV-pre-exposure prophylaxis (PrEP) among men who have sex with men in Switzerland: Testing an extended explanatory model drawing on the unified theory of acceptance and use of technology (UTAUT). Z Gesundh Wiss 2018; 26:247–259. 29780687 10.1007/s10389-017-0869-1 PMC5948261

[21] Bil JP Davidovich U van der Veldt WM, What do Dutch MSM think of preexposure prophylaxis to prevent HIV-infection? A cross-sectional study. AIDS 2015; 29:955–964. 25915169 10.1097/QAD.0000000000000639

[22] Coyer L van Bilsen W Bil J, Pre-exposure prophylaxis among men who have sex with men in the Amsterdam Cohort Studies: Use, eligibility, and intention to use. PLoS One 2018; 13:e0205663. 30312336 10.1371/journal.pone.0205663 PMC6185853

